# Takotsubo Cardiomyopathy as a Delayed Complication with a Herbicide Containing Glufosinate Ammonium in a Suicide Attempt: A Case Report

**DOI:** 10.1155/2012/630468

**Published:** 2012-11-19

**Authors:** Keiichiro Tominaga, Manabu Izumi, Masayuki Suzukawa, Takafumi Shinjo, Yoshimitsu Izawa, Chikara Yonekawa, Masaki Ano, Keisuke Yamashita, Tomohiro Muronoi, Reiko Mochiduki

**Affiliations:** ^1^Department of Emergency and Critical Care Medicine, Jichi Medical University, Tochigi 329-0498, Japan; ^2^Department of General Medicine, Saiseikai Utsunomiya Hospital, 911-1 Takebayashi, Utsunomiya City 321-0974, Japan

## Abstract

*Background*. Glufosinate ammonium has a famous delayed complication as respiratory failure, however, delayed cardiogenic complication is not well known. *Objectives*. The aim of this study is to report a takotsubo cardiomyopathy as a delayed complication of glufosinate ammonium for suicide attempt. *Case Report*. A 75-year-old woman ingested about 90 mL of Basta, herbicide for suicide attempt at arousal during sleep. She came to our hospital at twelve hours after ingesting. She was admitted to our hospital for fear of delayed respiratory failure. Actually, she felt down to respiratory failure, needing a ventilator with intubation at 20 hours after ingesting. Procedure around respiratory management had smoothly done with no delay. Her vital status had been stable, however, she felt down to circulatory failure and diagnosed as Takotsubo cardiomyopathy at about 41 hours after ingestion. There was no trigger activities or events to evoke mental and physical stresses. *Conclusion*. We could successfully manage takotsubo cardiomyopathy resulted in circulatory failure in a patient with glufosinate poisoning for suicide attempt. Takotsubo cardiomyopathy should be taken into consideration if circulatory failure is observed for unexplained reasons.

## 1. Introduction

The Basta herbicide (Bayer, Germany), containing glufosinate ammonium (18.5%) and an anionic surfactant, polyoxyethylene alkylether sulfate (30%), is widely used in many countries since 1984 [1]. Most of the cases with glufosinate poisoning were reported from Japan. most of glufosinate poisoning was occupied with Basta for a suicide attempt.

Glufosinate is a structural analogue of glutamic acid, a typical excitatory amino acid in the central nervous system (CNS), the main target of acute glufosinate poisoning, although the underlying cellular and molecular mechanisms of this action are not understood clearly. Glufosinate is thought to inhibit glutamine synthetase and glutamine decarboxylase, resulting in decreased glutamic acid levels and CNS symptoms (drowsiness, memory impairment, and seizures). A majority of this class of herbicides contain an anionic surfactant that increases blood permeability, resulting in decreasing circulatory blood volume, cardiac function, and resistance of systemic peripheral vessels [2]. Furthermore, some reports have linked glyphosate and its surfactant with mitochondrial impairment because of the structural similarity between glyphosate and glufosinate [3–5].

Takotsubo cardiomyopathy has a character of transient, reversible left ventricular dysfunction with normal coronary arteries [6]. Acute physical or emotional distress is thought to play a role in the development of Takotsubo cardiomyopathy through spike-like sympathetic stimulation. The prognosis for this entity is quite favorable with supportive care. We present a case of Takotsubo cardiomyopathy as a delayed complication with a herbicide containing glufosinate ammonium in a suicide attempt.

## 2. Case Report

A 75-year-old woman ingested about 90 mL of Basta herbicide for suicide attempt at arousal during sleep. She had vomited a lot: however, she fell asleep without telling someone else about it. Two hours later, she got up by herself, and she could not walk without supports for her dizziness. 

**Figure 1:**
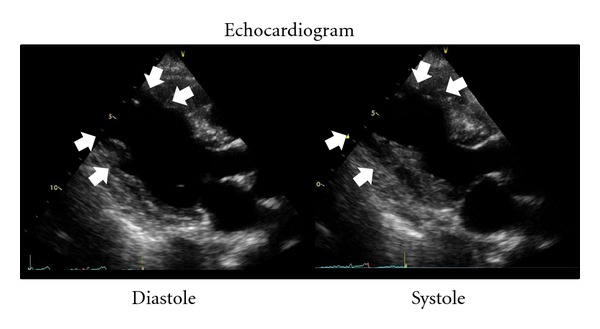
These images show echocardiography obtained at circulatory failure (41 hours after taking Basta) through cardiac cycle. There is akinesis lesion from mid to apex of left ventricle (LV) through cardiac cycle (white arrow). Meanwhile, there is hyperkinetic motion at base of LV.

**Figure 2:**
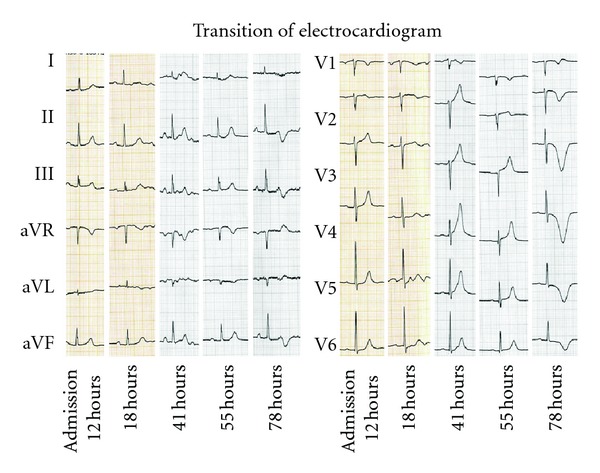
These images show transition of electrocardiogram. Times after ingestion of glufosinate were set as horizontal axes. “41 hours” means the time at onset of Takotsubo cardiomyopathy. Transit ST segment elevation and typical negative T wave were seen in precordial leads through clinical course.

Thus, she came to our hospital twelve hours after ingesting Basta. Her consciousness was alert, and therapeutic time window of gastric decontamination was over. But we, she, and her family did not give out what she ingested accurately. So we waited to make sure of the name of agrichemical. Not surprisingly, we made sure that she ingested “Basta,” containing glufosinate ammonium and decided her admission to our hospital to observe for delayed respiratory failure. Actually, she felt down to respiratory failure, needing a ventilator with intubation at 20 hours after ingesting. Procedures around respiratory management were smoothly done with no delay. Her vital status had been stable: however, she felt down to congestive heart failure at until 41 hours after ingestion. We did not use any catecholamine before this event. There were no trigger activities or events to evoke mental and physical stresses. The electrocardiography showed ST elevation in precordial leads (V2-6). The echocardiography revealed focal asynergy of left ventricle, similar to Takotsubo cardiomyopathy (Figures 1 and 2). However, we could not deny that ischemic heart attack caused this circulatory failure. We decided to examine her coronary arteries. However, the coronary angiography revealed neither significant stenosis nor occlusion of her coronary artery. After a while, her circulatory failure was improved with human atrial natriuretic peptide (carperitide). After that, she had another complication as aspiration pneumonia. It took two weeks to extubate and to achieve self-sustaining breathing without ventilator: however, she could move to the hospital for rehabilitation with wheel chair to achieve walking without any support.

## 3. Discussion

In Japan, it is famous that “Basta” herbicide containing glufosinate ammonium can cause delayed respiratory failure [2]. However, toxiosity of “Basta” is subjected to both glufosinate and an anionic surfactant, polyoxyethylene alkylether sulfate (AE). AE has toxicity for circulatory failure directly and indirectly [6]. Koyama et al. reported that AE itself reduces cardiac function, decreasing heart rate and blood pressure without calcium channel effects. Furthermore, AE also reduced arterial tonus through endothelial mechanism related with the concentration of AE, empirically [7, 8].

Basta is well known for many complications at some stages. In the early stage, many patients taking Basta have gastrointestinal symptoms (vomiting, nausea, and diarrhea). Afterwards, they gradually have the neurological and circulatory symptoms, such as seizure, convulsion, circulatory failure, and impaired respiration. Tajima et al. reported Takotsubo cardiomyopathy triggered by systemic convulsion in patient with glufosinate ammonium [9]. However, there is no report about takotsubo cardiomyopathy in patient with glufosinate ammonium as delayed complication simply.

In this case, there was no trigger event as physical and emotional distress around onset time. Respiratory failure which required ventilation occurred over 20 hours before the onset of takotsubo cardiomyopathy. Her disturbance of consciousness had been continued without fluctuation. We thought that this event was brought by glufosinate ammonium directly. About half of takotsubo cardiomyopathies have no emotional stress before onset: however, “attempting suicide” must be a trigger of emotional stress in this case. Meanwhile, the onset of cardiogenic shock occurred over 41 hours after attempting suicide, under the management of ventilator. Her emotional stress must be a peak just after attempting suicide and be getting lower with time. Thus, in this case, her takotsubo cardiomyopathy might be brought not only by emotional stress as attempting suicide but also by glufosinate itself. There was no direct relationship: however, we believe that there is a causal relationship between Basta and the development of takotsubo cardiomyopathy.

Michael RG reported the review of takotsubo cardiomyopathy with fluorouracil that 9 cases (4 males and 5 females) were evoked both with bolus infusion and continuous infusion from 2000 to 2011 [10]. They did not have emotional and physical distress. Thus, they reflect that fluorouracil has affinity to the heart as well.

We could successfully manage takotsubo cardiomyopathy resulted in circulatory failure associated with respiratory failure in a patient with glufosinate poisoning for suicide attempt. Takotsubo cardiomyopathy should be taken into consideration if circulatory failure is observed for unexplained reasons.
